# The ecdysone-induced bZIP transcription factor MafB establishes a positive feedback loop to enhance vitellogenesis and reproduction in the *Aedes aegypti* mosquito

**DOI:** 10.1073/pnas.2411688122

**Published:** 2025-01-10

**Authors:** Jia-Lin Wang, Zi-Qian Zhong, Ya-Zhou He, Jun-Hua Tian, Yu-Feng Wang, Alexander S. Raikhel

**Affiliations:** ^a^Hubei Key Laboratory of Genetic Regulation and Integrative Biology, School of Life Sciences, Central China Normal University, Wuhan 430079, China; ^b^Department of Entomology, University of California, Riverside, CA 92521; ^c^Institute of Integrative Genomic Biology, University of California, Riverside, CA 92521; ^d^College of Plant Protection, Nanjing Agricultural University, Nanjing 210095, China; ^e^Wuhan Center for Disease Control and Prevention, Wuhan 430022, China

**Keywords:** MafB, CncC, shade, vitellogenin, 20-hydroxyecdysone

## Abstract

Female mosquitoes require a vertebrate blood meal to activate reproduction, transmitting numerous devastating human diseases. Vitellogenesis is a central event of female reproduction that involves the massive production of vitellogenin (Vg) in the fat body and the maturation of ovaries. This process is controlled by the steroid hormone 20-hydroxyecdysone (20E); however, its molecular regulatory basis remains not completely understood. We found that the expression of *Aedes aegypti muscle aponeurosis fibromatosis B* (*AaMafB*), coding for a basic leucine zipper (bZIP) transcription factor, was significantly up-regulated after a blood meal. The 20E-bound ecdysone receptor–ultraspiracle heterodimer directly targeted the ecdysone response element in the promoter of *AaMafB*, activating its transcription. Coimmunoprecipitation assays illustrated the interaction between *Aa*MafB and Cap “n” collar C (*Aa*CncC), another bZIP transcription factor. RNA interference–mediated depletion of *Aa*MafB or *Aa*CncC led to impaired ovarian growth, decreased expression of *AaVg* and Halloween genes, and reduced 20E levels. The *Aa*MafB–*Aa*CncC heterodimer directly activated the transcription of *AaVg* and *AaShade* by targeting the antioxidant response element in their promoters. Together, our results indicate that *AaMafB* functions as an early 20E response gene, the product of which heterodimerizes with *Aa*CncC to maintain high 20E levels and facilitates activation of *AaVg* in mosquitoes after a blood meal.

## Significance

Female mosquitoes that serve as disease vectors require vertebrate blood to produce eggs. In *Aedes aegypti*, blood-feeding signals the brain to release neurohormones that trigger 20-hydroxyecdysone (20E) production. 20E is essential for vitellogenin (*Aa* Vg) activation and sustained high expression. We identified *muscle aponeurosis fibromatosis B* (*AaMafB*) as an early ecdysone-response gene that significantly increases its transcript abundance in *A. aegypti* following a blood meal. Silencing *Aa* MafB decreased 20E levels, reduced expression of Halloween genes and *AaVg*, and impaired ovarian growth. *Aa* MafB heterodimerized with *Aa* CncC, directly activating the transcription of *AaShade* and *AaVg*. Thus, *Aa* MafB mediates a positive feedback loop that amplifies 20E signaling and regulates vitellogenesis in *A. aegypti* after a blood meal.

Female hematophagous mosquitoes transmit pathogens of many human diseases and are responsible for nearly one million deaths annually, making them the deadliest animals in the world. The *Aedes aegypti* mosquito is a vector of Dengue, Yellow fever, chikungunya, and Zika viruses, which greatly burden human health and well-being (1–4). An incredible reproductive capacity is one of the reasons insect species thrive on earth. The massive synthesis of the primary yolk protein precursor (YPP)—vitellogenin (Vg)—in the fat body (FB) and its subsequent internalization into developing oocytes are essential for successful reproduction (5). In most insects, vitellogenesis is primarily governed by the sesquiterpenoid juvenile hormone (JH); in Dipteran mosquitoes, it is regulated by the steroid hormone 20-hydroxyecdysone (20E) (6). Deciphering the molecular mechanisms of blood-meal-triggered vitellogenesis and its endocrine control will help to develop innovative approaches for controlling mosquito populations and preventing mosquito-borne diseases.

Consumption of a blood meal stimulates the release of insulin-like peptides and ovary ecdysteroidogenic hormone from brain neurosecretory cells. These hormones then prompt the ovaries (OVs) to secrete ecdysteroids (7–9). 20E exerts its genomic function through the ecdysone receptor (EcR) and ultraspiracle (USP), an ortholog of the vertebrate retinoid X receptor (10). The 20E-bound EcR–USP heterodimer directly binds to the ecdysone response element (EcRE), activating the transcription of early-response genes, such as *Broad*-*Complex* (*BrC*), *ecdysone*-*induced protein 74* (*E74*), and *ecdysone*-*induced protein 75* (*E75*) (11–14). The products of these early genes, known as transcription factors, further activate many late target genes, amplifying the 20E signaling (15).

In the mosquito *A. aegypti*, the 20E titer rises sharply during the 12 to 18 h post-blood meal (PBM), peaking at 18 to 20 h PBM (16). Elevated 20E directly initiates *AaVg* expression by binding the 20E–*Aa*EcR–*Aa*USP complex to an EcRE in the *AaVg* promoter (17). The products of 20E early or early-late genes—acting synergistically with the *Aa*EcR–*Aa*USP heterodimer in different ways—lead to a massive expression of *AaVg* (18–20). Given that *AaVg* expression in response to 20E occurs in a stage- and tissue-specific manner, it is likely that other as-yet-uncharacterized factors and mechanisms are involved in fine-tuning the regulation of this process.

The small muscle aponeurosis fibromatosis (sMaf) is a basic leucine zipper (bZIP) transcription factor (21). The sMaf forms a heterodimer with the nuclear factor erythroid-derived 2-like 2 (Nrf2). On exposure to xenobiotics or oxidative stress, Nrf2 dissociates from Kelch-like ECH-associated protein-1 in the cytoplasm and translocates to the nucleus (21–24). Additionally, the insect Cap “n” collar isoform C (CncC), which is the ortholog of the vertebrate protein Nrf2, also functions as a bZIP transcription factor (25). The sMaf–CncC heterodimer activates detoxification or antioxidant genes harboring the antioxidant response element (ARE) in the 5′-end regulatory regions (26, 27). While extensive research has been conducted on the role of the sMaf-CncC heterodimer in detoxification or antioxidant processes (28, 29), its involvement in reproduction remains obscure. Recent findings have demonstrated that CncC mediates the trade-off between detoxification and reproduction by transcriptionally regulating genes involved in detoxification and hormone signaling pathways (30).

Our previous transcriptomic analyses indicated strong upregulation of *AaMafB* (*AAEL007686*) in female *A. aegypti* after a blood meal, suggesting its potential involvement in blood-meal-activated reproductive events (31). This study demonstrated that *AaMafB*, as an early gene, is implicated in the ecdysteroid hierarchy. *Aa*MafB directly activated the expression of *AaShade* (*AaShd*, *CYP314A1*) and *AaVg* by heterodimerizing with *Aa*CncC (AAEL005077) and targeting the ARE in their promoters. Our findings characterized *AaMafB* as a 20E early gene and revealed a mechanism through which *Aa*MafB modulates reproductive events in the *A. aegypti* mosquito.

## Results

### *AaMafB* Expression in the FB and OV of Adult Female Mosquitoes During the Vitellogenic Phase.

**Fig. 1.**
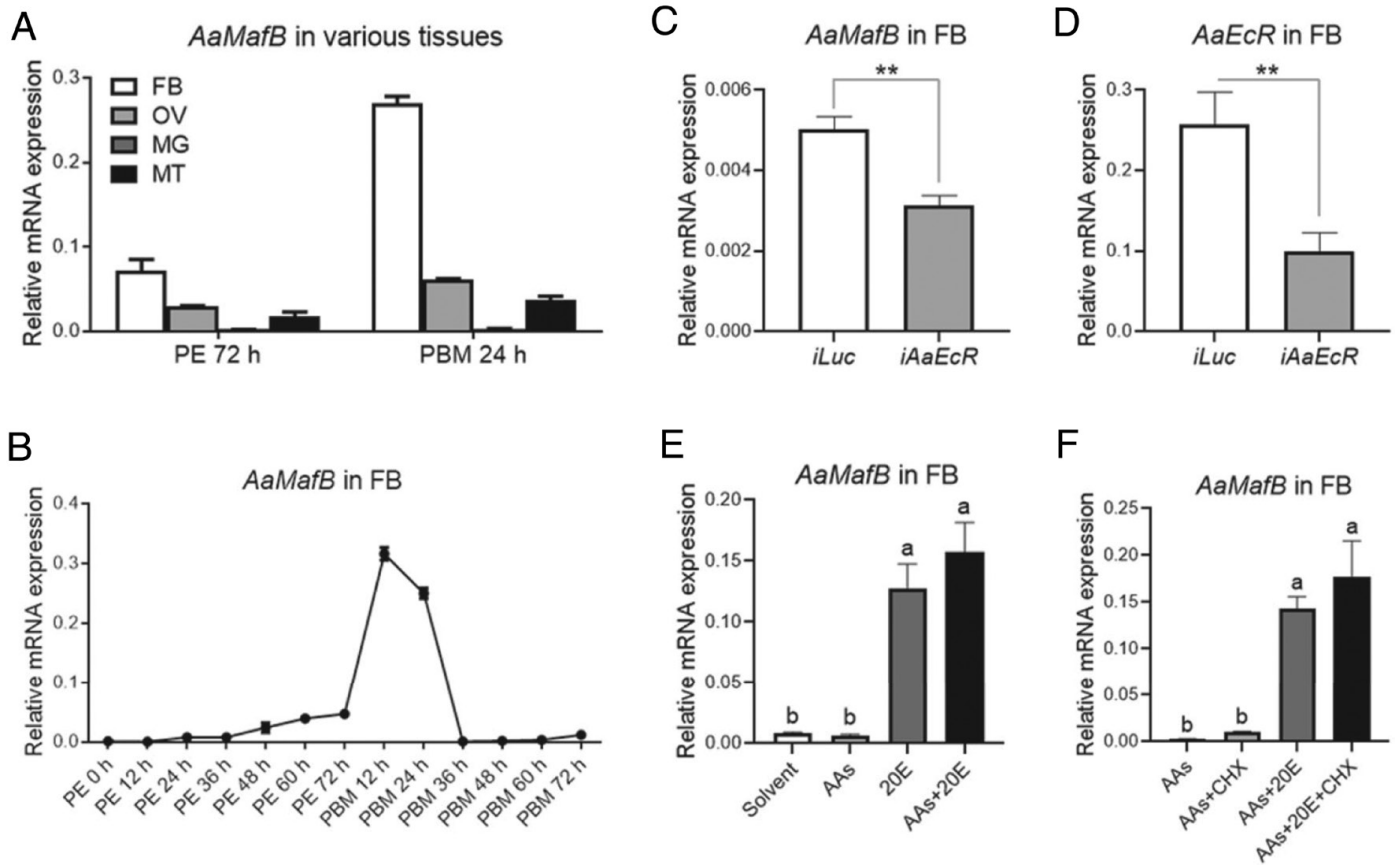
20E and its receptor *Aa*EcR induce the expression of *AaMafB* directly. (*A*) Relative mRNA levels of *AaMafB* in various tissues. Samples were collected from the FB, ovary (OV), midgut (MG), and Malpighian tubules (MT) of female *A. aegypti* at 72 h PE and 24 h PBM. (*B*) Relative mRNA levels of *AaMafB* in the FB of female *A. aegypti* at various developmental stages. FBs were collected at 0, 12, 24, 36, 48, 60, and 72 h PE and 12, 24, 36, 48, 60, and 72 h PBM. (*C*) Suppressed expression of *AaMafB* in *Aa*EcR-depleted (*iAaEcR*) FB of female *A. aegypti*. (*D*) RT-qPCR confirming the silencing of *AaEcR* in FB. *iLuc*, *luciferase* dsRNA-injected. (*E*) Relative mRNA levels of *AaMafB* in the FB collected from female mosquitoes at 96 h PE and incubated in culture medium containing either ethanol as solvent control or amino acids (AAs), 20E or both AAs and 20E (AAs+20E) dissolved in ethanol. (*F*) Relative mRNA levels of *AaMafB* in the FB collected from female mosquitoes at 96 h PE and incubated in a culture medium containing AAs, AAs+CHX, AAs+20E, or AAs+20E + CHX. Asterisks indicate statistically significant differences determined by a two-tailed Student’s *t* test (**P < 0.01). Different letters (a and b) above the bars indicate statistically significant differences based on a one-way ANOVA test (*P* < 0.0001).

To validate the elevated expression of *AaMafB* during the vitellogenic phase detected by transcriptomic analyses (31), we further analyzed its expression using quantitative real-time PCR (RT-qPCR). The *AaMafB* transcript was expressed predominantly in the FB of adult female mosquitoes (Fig. 1*A*). In the FB, the *AaMafB* transcript exhibited low levels of expression from 0 h to 72 h post-eclosion (PE), significantly increased at 12 h and 24 h PBM, and subsequently declined sharply at 36 h PBM (Fig. 1*B*). The abundance of *AaMafB* transcripts in the OV peaked at 12 h PBM (*SI Appendix*, Fig. S1*A*), similar to that in the FB. These *AaMafB* transcription profiles correlated with the endogenous 20E titer (16), suggesting a rapid responsiveness of *AaMafB* to 20E.

### The 20E–*Aa*EcR–*Aa*USP Complex Directly Activates the Transcription of *AaMafB*.

As the *AaMafB* expression profiles correlate with the endogenous 20E titer, we wondered whether 20E and its receptor *Aa*EcR controlled *AaMafB* expression. RT-qPCR analyses indicated that *AaMafB* transcripts were significantly lower in RNAi-mediated *Aa*EcR-depleted FB (Fig. 1*C*). The efficiency of *AaEcR* RNAi depletion was confirmed (Fig. 1*D*). To determine whether the action of *Aa*EcR on the expression of *AaMafB* is direct or mediated by an intermediate factor, we conducted in vitro FB culture assays. The expression level of *AaMafB* was significantly elevated by 20E but not by AAs (Fig. 1*E*). Cycloheximide (CHX), a protein biosynthesis inhibitor, did not suppress 20E-mediated induction of *AaMafB* (Fig. 1*F*), indicating that 20E induced the expression of *AaMafB* directly.

**Fig. 2.**
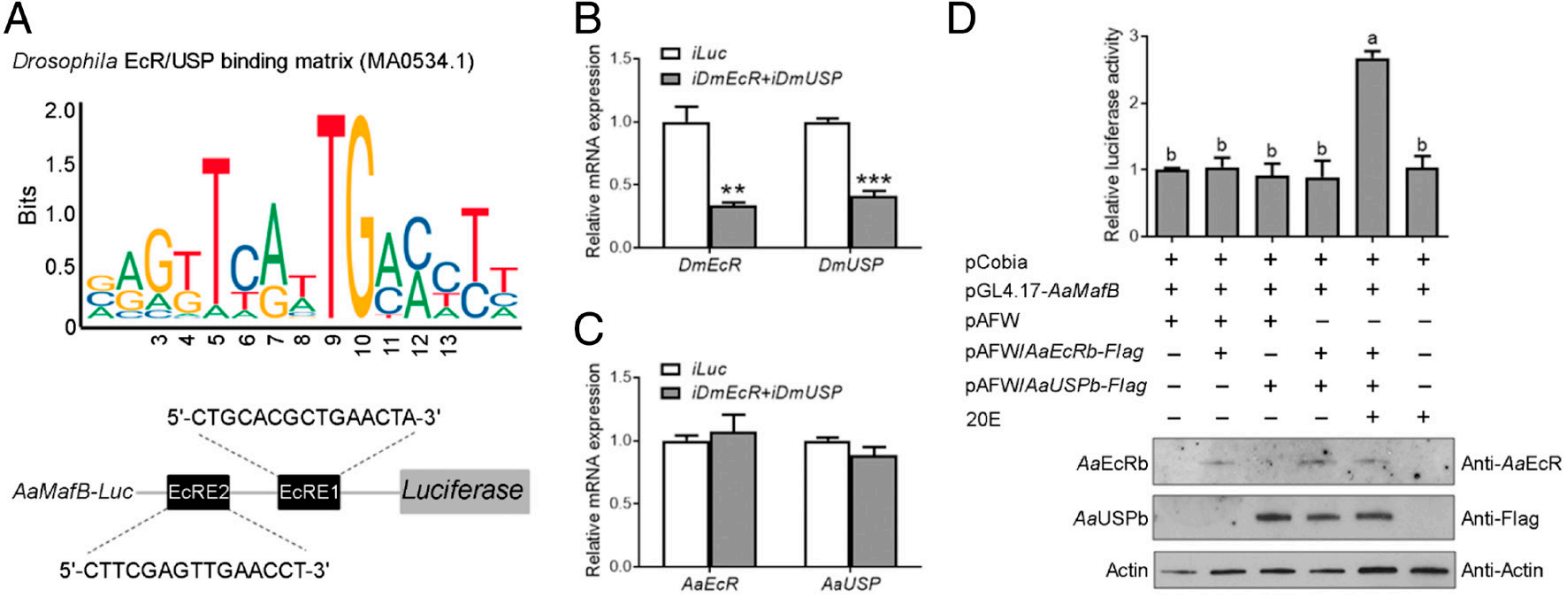
*AaMafB* is transcriptionally regulated by the 20E–*Aa*EcR–*Aa*USP complex. (*A*) A luciferase reporter pGL4.17-*AaMafB* construct carrying predicted EcRE1 and EcRE2. The predicted EcREs were searched against the *Drosophila* EcR–USP binding matrix (MA0534.1) using the JASPAR online server (https://jaspar.genereg.net/). (*B*) The efficiency of *D. melanogaster EcR* (*DmEcR*) and *usp* (*DmUSP*) depletion in S2 cells after treatment with *DmEcR* and *DmUSP* dsRNAs. (*C*) No significant variation in mosquito *AaEcRb-Flag* or *AaUSPb-Flag* mRNA levels in S2 cells after treatment with *DmEcR* and *DmUSP* dsRNAs. *iLuc* was used as a control. All expressions are calculated against *Drosophila β-actin*. (*D*) Dual-luciferase reporter assays show the transcriptional activation of the *AaMafB* promoter by *Aa*EcRb–*Aa*USPb in the presence of 20E. S2 cells were cotransfected with the reporter vector pGL4.17-*AaMafB*, the overexpression plasmids pAFW/*AaEcRb-Flag* and/or pAFW/*AaUSPb-Flag*, and the pCopia plasmid. The expression of *Aa*EcRb-Flag and/or *Aa*USPb-Flag in S2 cells was detected by immunoblotting using antibodies against *Aa*EcR or Flag. β-actin was used as a loading control. Asterisks indicate statistically significant differences determined by a two-tailed Student’s *t* test (**P < 0.01 and ***P < 0.001). Different letters (a and b) above the bars indicate statistically significant differences based on a one-way ANOVA test (*P* < 0.0001).

EcR interacts with its heterodimeric partner USP in the presence of 20E and acts on the EcRE to activate the transcription of target genes (32, 33). We performed a dual-luciferase reporter assay to investigate whether *Aa*EcR directly regulates *AaMafB* transcription. We cloned the upstream regulatory sequence of *AaMafB* (nt -1217 to -729), which contains two predicted EcREs (Fig. 2*A*), into the pGL4.17 vector. To minimize the effect of endogenous *Drosophila melanogaster* EcR (DmEcR) and USP (DmUSP) in S2 cells, dsRNA targeting *DmEcR* or *DmUSP* was synthesized. The expression of *DmEcR* and *DmUSP* was significantly suppressed in S2 cells pretreated with *DmEcR* and *DmUSP* dsRNAs (Fig. 2*B*). At the same time, the transcripts of transfected mosquito *AaEcRb-Flag* or *AaUSPb-Flag* were not affected (Fig. 2*C*). Overexpression of *Aa*EcRb or *Aa*USPb alone did not induce luciferase reporter activity. Coexpression of *Aa*EcRb and *Aa*USPb significantly activated reporter activity only in the presence of 20E (Fig. 2*D*), suggesting that the 20E–*Aa*EcR–*Aa*USP complex is required for *AaMafB* transcription. Overexpression of *Aa*EcRb and/or *Aa*USPb was confirmed by immunoblotting (Fig. 2*D*).

To determine whether the 20E–*Aa*EcR–*Aa*USP complex physically interacts with the *AaMafB* promoter, we conducted an electrophoretic mobility shift assay (EMSA) using nuclear extracts from S2 cells expressing *Aa*EcRb-Flag and *Aa*USPb-Flag in the presence of 20E. A dense band was visualized in samples incubated with the labeled *AaMafB* probe 1 (covering the EcRE1), which was abolished after preincubation with an excess of the unlabeled *AaMafB* probe 1, suggesting the specificity of the protein–DNA interaction. The band was also abolished after preincubation of nuclear extracts with anti-Flag or anti-*Aa*EcR antibody, suggesting the presence of *Aa*EcRb-Flag and *Aa*USPb-Flag in the binding complex (*SI Appendix*, Fig. S2*A*). The band was diminished mainly after mutation of the motif (TGAAC) to (GTCTA). It was also diminished after mutation of nucleotides before the motif (TGAAC). When this mutation was accumulated, and the sequence was similar to EcRE2 (covered by *AaMafB* probe 2), the band almost disappeared, consistent with the performance of *AaMafB* probe 2 (*SI Appendix*, Fig. S2*B*). These results indicate that the motif (TGAAC) and the six-nucleotide sequence (GCACGC) preceding it determine the binding capacity of the EcRE in the *AaMafB* promoter.

### *Aa*MafB Is Essential for Vitellogenesis and Reproduction in Adult Female Mosquitoes.

**Fig. 3.**
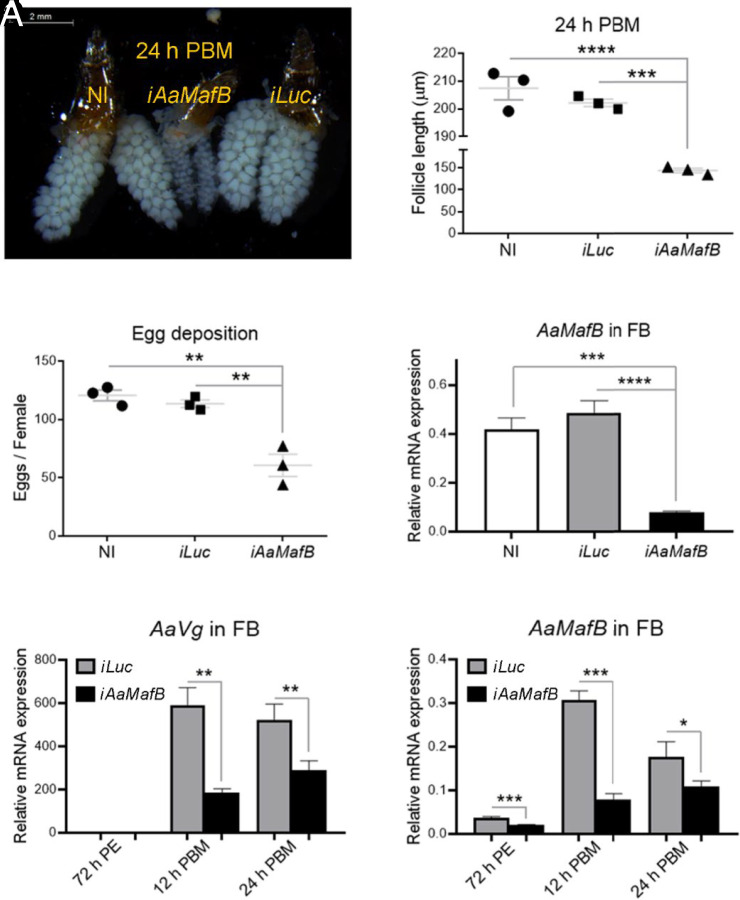
Depletion of *Aa*MafB decreases follicle size, egg number, and *AaVg* expression. (*A*) Representative OVs at 24 h PBM. (*B*) Average follicle length at 24 h PBM. (*C*) Average egg numbers deposited by female mosquitoes. (*D*) RT-qPCR confirming the depletion of *AaMafB* in the FB. (*E*) A dramatic decline in *AaVg* transcripts in *Aa*MafB-depleted FB detected using RT-qPCR. (*F*) RT-qPCR showing the efficiency of *AaMafB* depletion in FB. NI, noninjected; *iLuc*, *luciferase* dsRNA-injected; *iAaMafB*, *AaMafB* dsRNA-injected. Statistical differences were determined using a two-tailed Student’s *t* test or one-way ANOVA test. Asterisks indicate significant differences (*P < 0.05, **P < 0.01, ***P < 0.001, and ****P < 0.0001).

To investigate the role of *Aa*MafB, we performed RNAi-mediated depletion of *Aa*MafB in newly emerged female mosquitoes, followed by evaluation of ovarian development at 24 h PBM. As shown in Fig. 3*A*, *Aa*MafB depletion impaired ovarian growth and oocyte maturation. Noninjected (NI) female mosquitoes and those injected with *luciferase* dsRNA (*iLuc*) were used as controls, with average primary follicle lengths of 207 μm and 202 μm, respectively. In contrast, the average primary follicle length in mosquitoes injected with *AaMafB* dsRNA (*iAaMafB*) was 144 μm, significantly smaller than that of the controls (Fig. 3*B*). The NI and *iLuc* controls laid an average of 121 and 113 eggs per female, respectively. Conversely, *iAaMafB* dramatically reduced fecundity, resulting in an average of only 61 eggs laid per female (Fig. 3*C*). Eggs from the *iLuc* control had a hatching rate of approximately 85%. In contrast, only about 0.03% of eggs from the *iAaMafB* group hatched (*SI Appendix*, Fig. S1*B*). The efficiency of *AaMafB* RNAi depletion was confirmed in the FB at 24 h PBM (Fig. 3*D*).

As a major YPP, Vg is synthesized in the FB, secreted into the hemolymph, and packaged in developing oocytes (5). To determine whether *Aa*MafB is involved in *Aa*Vg synthesis, newly emerged female mosquitoes were treated with dsRNAs, and FBs from various stages were collected. The expression of *AaVg* was scarcely detectable at 72 h PE. During mosquito vitellogenesis, *AaVg* was transcribed in large quantities. Depletion of *Aa*MafB significantly suppressed the expression of *AaVg* at 12 h and 24 h PBM (Fig. 3*E*). The expression of *AaVg receptor* (*AaVgR*) in the OV, responsible for the endocytosis of *Aa*Vg (5), was also lower after *Aa*MafB depletion (*SI Appendix*, Fig. S1*C*). The efficiency of *AaMafB* RNAi depletion at various stages was confirmed using RT-qPCR (Fig. 3*F*).

### *Aa*MafB Interacts with a bZIP Transcription Factor *Aa*CncC to Promote Vitellogenesis and Reproduction.

**Fig. 4.**
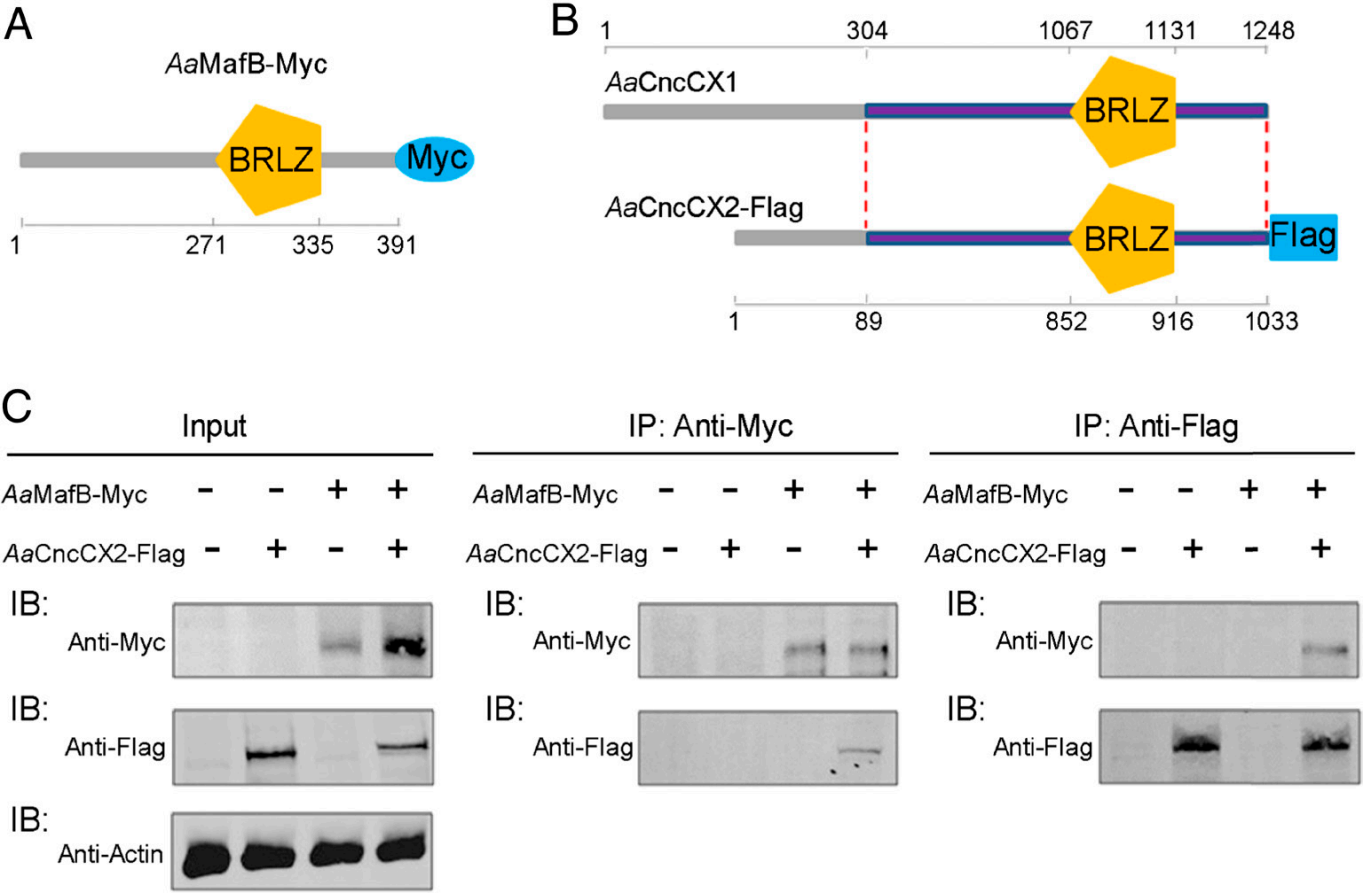
Interaction between *Aa*MafB and *Aa*CncC. (*A* and *B*) Schematic diagram of *Aa*MafB-Myc (*A*) and *Aa*CncCX2-Flag (*B*) fusion protein expressed in S2 cells. *Aa*CncCX1 and *Aa*CncCX2 share a conserved C-terminal region (between the red dotted lines). BRLZ, basic region leucine zipper. (*C*) The *Aa*MafB–*Aa*CncCX2 interaction analyzed by reciprocal co-IP. Proteins were expressed in S2 cells. Immunoprecipitation (IP) was performed using anti-Myc or anti-Flag antibody overnight at 4 °C. Immunoprecipitants were detected using immunoblotting (IB) with the indicated antibodies. Antibody against β-actin was used as a loading control.

*Aa*MafB, a bZIP transcription factor, contains a basic region leucine zipper (BRLZ) domain (Fig. 4*A*) and belongs to the sMaf family. The sMaf proteins have been reported to interact with Nrf2, which is orthologous to CncC (25). *Aa*CncC has been characterized in *A. aegypti* (34) and has two isoforms, *Aa*CncCX1 (XP_021693905) and *Aa*CncCX2 (XP_021693906). Both isoforms share an identical C-terminal BRLZ domain (Fig. 4*B*) that mediates sequence-specific DNA binding and dimerization (35). Given that *Aa*CncCX1 and *Aa*CncCX2 share a conserved C terminus with an included BRLZ domain, we selected the shorter to test whether it interacts with *Aa*MafB. The entire lengths of *Aa*MafB (amino acid 1 to 391) and *Aa*CncCX2 (amino acid 1 to 1,033) were expressed in S2 cells, with a C-terminal Myc and Flag tag, respectively (Fig. 4 *A* and *B*). Reciprocal coimmunoprecipitation (co-IP) assays showed that Flag-tagged *Aa*CncCX2 could only be detected in the immunoprecipitants of Myc-tagged *Aa*MafB, and vice versa (Fig. 4*C*). These results demonstrated the interaction between *Aa*MafB and *Aa*CncC.

### Depletion of *Aa*CncC Impairs Egg Development and Decreases *AaVg* Expression.

Since *Aa*CncC was identified as an *Aa*MafB-interacting protein, we sought to determine whether it plays a role in egg development. Considering that five *AaCncC* transcripts encode *Aa*CncC isoforms, we designed a pair of primers based on the conserved region to quantify the total *AaCncC* transcripts. RT-qPCR analyses indicated that the abundance of *AaCncC* transcripts in the FB was slightly lower at 12 h PBM than at 72 h PE and then elevated again in 24 h PBM samples (*SI Appendix*, Fig. S3*A*). In vitro FB cultural assays showed that 20E did not significantly affect the expression of *AaCncC* (*SI Appendix*, Fig. S3*B*). Further, we analyzed the phenotypic manifestations in *Aa*CncC-depleted female mosquitoes by injecting the dsRNA targeting the common region of *AaCncC* transcripts. The ovarian growth at 24 h PBM was dramatically arrested after *Aa*CncC depletion (*SI Appendix*, Fig. S3*C*). The average length (142 μm) of primary follicles in *Aa*CncC-depleted mosquitoes was much smaller than that in the NI and *iLuc* controls, with average lengths of 215 μm and 219 μm, respectively (*SI Appendix*, Fig. S3*D*). In addition, *Aa*CncC depletion significantly suppressed the expression of *AaVg* at 24 h PBM (*SI Appendix*, Fig. S3*E*). The efficiency of *AaCncC* RNAi depletion in FB at 24 PBM was confirmed using RT-qPCR (*SI Appendix*, Fig. S3*F*).

### *Aa*MafB–*Aa*CncC Heterodimer Directly Activates the *AaVg* Transcription.

**Fig. 5.**
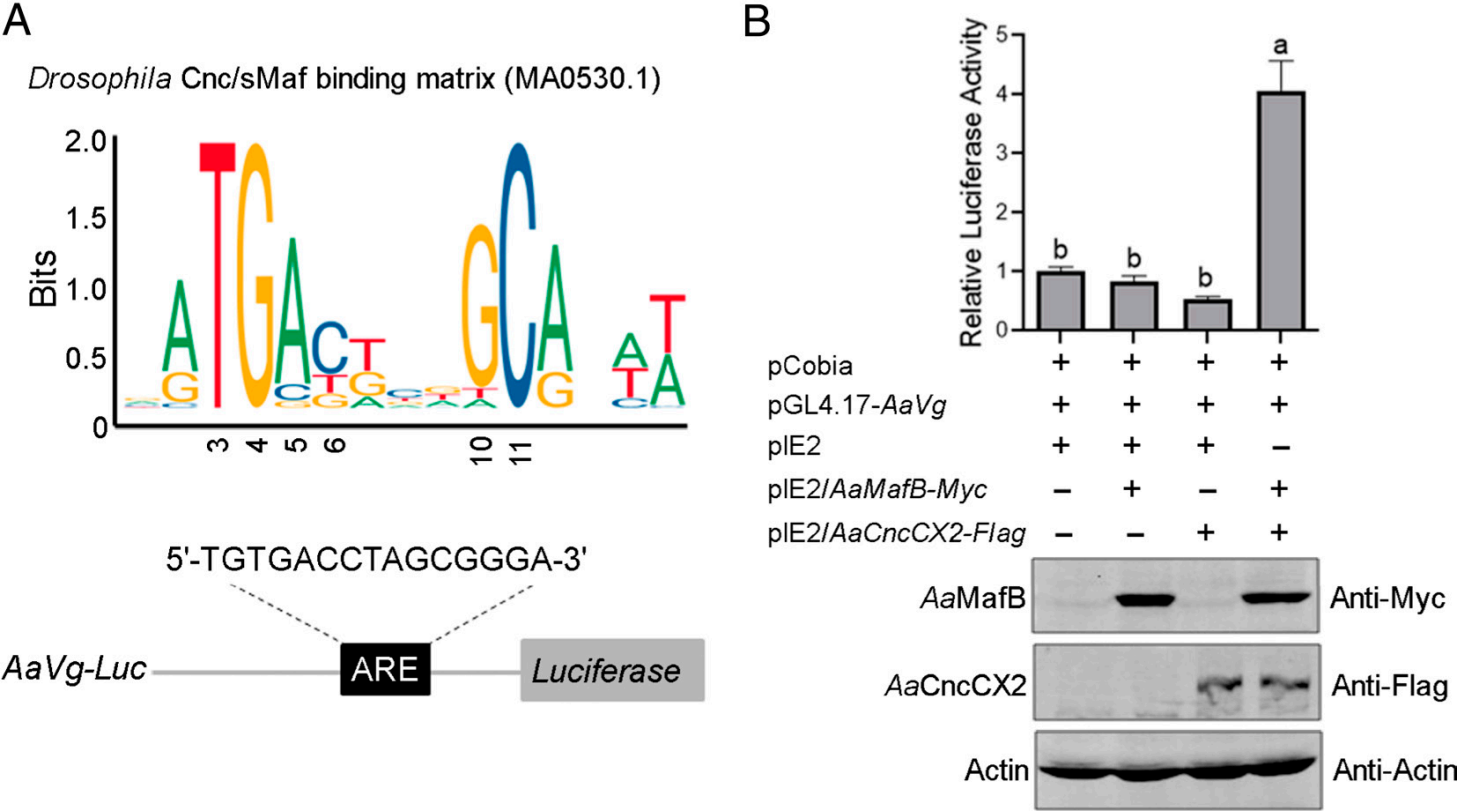
*AaVg* is transcriptionally regulated by the *Aa*MafB–*Aa*CncC heterodimer. (*A*) A luciferase reporter pGL4.17-*AaVg* construct carrying a predicted ARE. The predicted ARE was searched against the *Drosophila* Cnc–sMaf binding matrix (MA0530.1) using the JASPAR online server (https://jaspar.genereg.net/). (*B*) Dual-luciferase reporter assays show the transcriptional activation of the *AaVg* promoter by the *Aa*MafB–*Aa*CncCX2 heterodimer. S2 cells were cotransfected with the reporter vector pGL4.17-*AaVg*, the overexpression plasmids pIE2/*AaMafB-Myc* and/or pIE2/*AaCncCX2-Flag*, and the pCopia plasmid. The expression of *Aa*MafB-Myc and/or *Aa*CncCX2-Flag in S2 cells was detected by IB using antibodies against Myc or Flag. β-actin was used as a loading control. Different letters (a and b) on the bars indicate significant differences based on a one-way ANOVA test (*P* < 0.0001).

Since *Aa*MafB and *Aa*CncC interact with each other and both are involved in *AaVg* expression, we wondered whether the *Aa*MafB–*Aa*CncC heterodimer directly activates the expression of *AaVg*. Nrf2 (Cnc)–sMaf heterodimer is known to bind to the ARE and activates the expression of cytoprotective genes (21). The Cnc–sMaf binding site with the core ARE sequence (TGACNNNGC) was predicted (Fig. 5*A*). We cloned the upstream regulatory sequence of *AaVg* (nt -1,023 to -26) with a predicted ARE into the pGL4.17 vector (Fig. 5*A*). A transient transfection assay indicated that overexpression of *Aa*MafB or *Aa*CncCX2 alone could not induce *AaVg* reporter activity. Coexpression of *Aa*MafB and *Aa*CncCX2 significantly activated reporter activity (Fig. 5*B*), suggesting that *Aa*MafB–*Aa*CncCX2 heterodimer is required for *AaVg* transcription. IB confirmed the overexpression of *Aa*MafB and/or *Aa*CncCX2 (Fig. 5*B*).

To determine whether the *Aa*MafB–*Aa*CncCX2 heterodimer physically interacts with the *AaVg* promoter, we performed EMSA using nuclear extracts from S2 cells expressing *Aa*MafB-Myc and *Aa*CncCX2-Flag. A dense band was visualized when the sample expressing *Aa*MafB-Myc, *Aa*CncCX2-Flag, or both were incubated with the labeled *AaVg* probe. In the latter, the band was abolished after preincubation with an excess of the unlabeled *AaVg* probe, demonstrating the specificity of the protein–DNA interaction. The band was diminished mainly after preincubation of nuclear extracts with anti-Myc or anti-Flag antibodies, suggesting the presence of *Aa*MafB-Myc and *Aa*CncCX2-Flag in the binding complex (*SI Appendix*, Fig. S4*A*). Additionally, the band disappeared after mutation of the core ARE motif or mutation of nucleotides before the core ARE motif, either preincubation of the sample expressing *Aa*MafB-Myc, *Aa*CncCX2-Flag, or both (*SI Appendix*, Fig. S4*B*). These observations suggest that the core ARE and the four-nucleotide motif (CGTG) determine the binding capacity of the *AaVg* promoter with *Aa*MafB homodimer, *Aa*CncCX2 homodimer, or *Aa*MafB–*Aa*CncCX2 heterodimer.

### The Lack of Synergistic Activation of *AaVg* Expression by the *Aa*MafB–*Aa*CncC and *Aa*EcR–*Aa*USP.

Given the modest activation of *AaVg* by the *Aa*MafB–*Aa*CncC (Fig. 5*B*), we explored the possibility of its synergistic interaction with other transcription factors. Considering the previously identified EcRE (17) and the ARE identified here in the *AaVg* promoter, we investigated whether the *Aa*MafB–*Aa*CncC heterodimer synergizes with the *Aa*EcR–*Aa*USP receptor to enhance *AaVg* expression. Therefore, the upstream regulatory sequence of *AaVg* (nt -1,023 to -26), containing both an identified EcRE and ARE, was cloned into the pGL4.17 vector (pGL4.17-*AaVg*, *SI Appendix*, Fig. S5*A*). A transient transfection assay showed that overexpression of *Aa*MafB–*Aa*CncCX2 led to a slight increase in reporter activity (~1.6 fold) in response to 20E (*SI Appendix*, Fig. S5*B*, lanes 1 and 2), suggesting that this induction might be due to endogenous DmEcR–DmUSP. Overexpression of *Aa*EcRb–*Aa*USPb resulted in a more substantial increase in reporter activity (~8.5 fold) in the presence of 20E (*SI Appendix*, Fig. S5*B*, lanes 3 and 4). Coexpression of *Aa*MafB–*Aa*CncCX2 and *Aa*EcRb–*Aa*USPb further increased reporter activity (~11.2 fold) after the 20E addition (*SI Appendix*, Fig. S5*B*, lanes 5 and 6).

To further validate these findings, we constructed pGL4.17-*AaVg* derivatives lacking specific regulatory elements: pGL4.17-*AaVg*ΔARE (lacking the ARE), pGL4.17-*AaVg*ΔEcRE (lacking the EcRE), and pGL4.17-*AaVg*ΔARE+ΔEcRE (lacking both the ARE and EcRE) (*SI Appendix*, Fig. S5*A*). These derivative plasmids were individually transfected. Coexpression of *Aa*MafB–*Aa*CncCX2 and *Aa*EcRb–*Aa*USPb activated reporter activity with the pGL4.17-*AaVg*ΔARE construct (~9.8 fold), but not with the pGL4.17-*AaVg*ΔEcRE or pGL4.17-*AaVg*ΔARE+ΔEcRE constructs (*SI Appendix*, Fig. S5*B*, lanes 5 and 6). These results indicate no apparent synergistic activation of the *AaVg* promoter by the *Aa*MafB–*Aa*CncC heterodimer and the *Aa*EcR–*Aa*USP receptor.

### *Aa*MafB–*Aa*CncC Heterodimer Directly Activates *AaShd* Transcription and Sustains High 20E Levels during the Vitellogenic Phase.

**Fig. 6.**
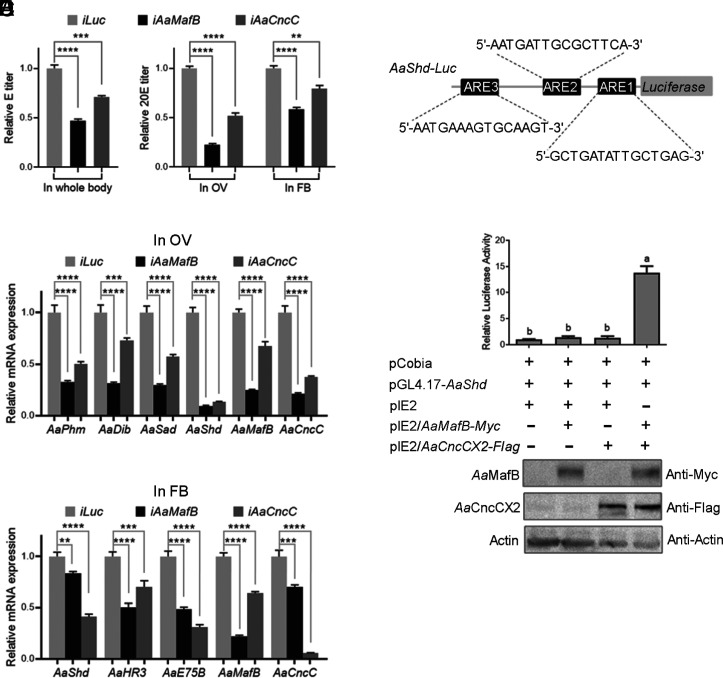
*AaShd* is transcriptionally regulated by the *Aa*MafB–*Aa*CncC heterodimer. (*A*) Depletion of *Aa*MafB or *Aa*CncC resulted in lower E and 20E titers at 24 PBM. Whole bodies of female *A. aegypti* were used for E measurement, whereas OVs and FBs were collected for 20E quantification. (*B* and *C*) Depletion of *Aa*MafB or *Aa*CncC led to lower expression levels of ecdysteroid pathway genes in OV (*B*) and FB (*C*). Samples were collected from *Aa*MafB-depleted (*iAaMafB*), *Aa*CncC-depleted *(iAaCncC*), and control (*iLuc*) female *A. aegypti* at 24 h PBM. (*D*) A luciferase reporter pGL4.17-*AaShd* construct carrying predicted ARE1, ARE2, and ARE3. The predicted AREs were searched against the *Drosophila* Cnc–sMaf binding matrix (MA0530.1). (*E*) Dual-luciferase reporter assays showing the transcriptional activation of the *AaShd* promoter by the *Aa*MafB–*Aa*CncCX2 heterodimer. S2 cells were cotransfected with the reporter vector pGL4.17-*AaShd*, the overexpression plasmids pIE2/*AaMafB-Myc* and/or pIE2/*AaCncCX2-Flag*, and the pCopia plasmid. The expression of *Aa*MafB-Myc and/or *Aa*CncCX2-Flag in S2 cells was detected by IB with antibodies against Myc or Flag. β-actin was used as a loading control. Asterisks indicate statistically significant differences determined by a one-way ANOVA test (**P < 0.01, ***P < 0.001, and ****P < 0.0001). Different letters (a and b) above the bars indicate statistically significant differences (*P* < 0.0001).

In comparison with *Aa*EcR–*Aa*USP, the direct role of the *Aa*MafB–*Aa*CncC heterodimer in activating *AaVg* expression appears to be less significant, with no observed synergistic effect (Fig. 5*B* and *SI Appendix*, Fig. S5). However, the phenotypic changes observed following the depletion of *Aa*MafB or *Aa*CncC suggest that the heterodimer regulates vitellogenesis through alternative mechanisms. Since CncC is known to directly activate the transcription of ecdysteroid biosynthetic genes (30, 36, 37), we propose that *Aa*MafB may also be involved as an interacting partner of *Aa*CncC. Indeed, depletion of *Aa*MafB or *Aa*CncC reduced ecdysone (E) titers in female *A. aegypti* at 24 h PBM and diminished 20E levels in the OV and FB (Fig. 6*A*).

The Halloween genes *Phantom* (*AaPhm*, *CYP306A1*), *Disembodied* (*AaDib*, *CYP302A1*), *Shadow* (*AaSad*, *CYP315A1*), and *Shade* (*AaShd*, *CYP314A1*), crucial for ecdysteroid biosynthesis (38), contain predicted Cnc–sMaf binding sites in their promoters (*SI Appendix*, Table S1). The adult OV of many insects, characterized by the high expression of Halloween genes, is a primary source of ecdysteroids (38, 39). We assessed expression levels of *AaPhm*, *AaDib*, *AaSad*, and *AaShd*, noting reduced transcripts in the OV of *Aa*MafB- or *Aa*CncC-depleted mosquitoes at 24 h PBM (Fig. 6*B*). Moreover, depletion of *Aa*MafB or *Aa*CncC inhibited *AaShd* expression in the FB concurrently, along with suppression of *hormone receptor 3* (*AaHR3*) and *AaE75B* (Fig. 6*C*), which are 20E early-response genes (33, 40). The efficiency of RNAi-mediated depletion of *AaMafB* or *AaCncC* in OV and FB was confirmed (Fig. 6 *B* and *C*).

The Shade facilitates the conversion of E to 20E in peripheral tissues (38, 41). We cloned the upstream regulatory sequence of *AaShd* (nt -1,600 to -336) containing three predicted AREs into the pGL4.17 vector (Fig. 6*D*). Overexpression of *Aa*MafB or *Aa*CncCX2 alone did not significantly activate reporter activity; however, their coexpression markedly enhanced reporter activity (Fig. 6*E*), indicating that the *Aa*MafB–*Aa*CncCX2 heterodimer is required for *AaShd* transcription. IB verified the overexpression of *Aa*MafB and/or *Aa*CncCX2 (Fig. 6*E*).

To further elucidate the physical interaction between the *Aa*MafB–*Aa*CncCX2 heterodimer and the *AaShd* promoter, EMSAs were performed using *AaShd* probes 1, 2, and 3, covering the corresponding AREs (*SI Appendix*, Fig. S6*A*). The EMSA results revealed stronger binding of the heterodimer to probes 1 and 3 than to probe 2, with specificity demonstrated by the abolition of bands upon preincubation with excess unlabeled probes (*SI Appendix*, Fig. S6*B*). These findings indicate that the *Aa*MafB–*Aa*CncC heterodimer directly activates *AaShd* transcription, likely contributing to the elevated 20E levels during the vitellogenic phase.

### Ectopic Application of 20E Partially Restores Ovarian Growth and *AaVg* Expression in *Aa*MafB- or *Aa*CncC-Depleted Female Mosquitoes.

To investigate whether the reduction in 20E levels caused by *Aa*MafB or *Aa*CncC depletion impacts vitellogenesis, we administered 20E into the hemocoel of mosquitoes with these depletions at 12 h PBM. By 24 h PBM, ovarian growth in both depletion groups showed partial restoration following the 20E injection, although the OVs remained smaller than those in *iLuc* plus solvent groups (*SI Appendix*, Fig. S7 *A* and B). In *Aa*MafB-depleted mosquitoes, the average length of primary follicles in the 20E-treated group (169 μm) was significantly greater than in the solvent control group (90 μm) (*SI Appendix*, Fig. S7*C*). A similar outcome was observed in *Aa*CncC-depleted mosquitoes, where the average follicle lengths were 177 μm in the 20E-treated group compared to 120 μm in the solvent control group (*SI Appendix*, Fig. S7*D*). Additionally, 20E treatment significantly up-regulated *AaVg* expression in the FB of either *Aa*MafB- or *Aa*CncC-depleted mosquitoes at 24 h PBM (*SI Appendix*, Fig. S7 *E* and F). These results suggest that 20E injection partially compensates for ovarian growth deficiencies caused by *Aa*MafB or *Aa*CncC depletion, reinforcing the role of *Aa*MafB–*Aa*CncC heterodimer in sustaining high 20E levels during the vitellogenic phase.

## Discussion

The bZIP transcription factors sMaf and CncC are established xenobiotic sensors, playing pivotal roles in detoxification and antioxidant responses (26, 27). For example, the sMaf–CncC heterodimer enhances the expression of detoxification enzyme CYPs, which is pivotal in conferring insecticide resistance (28, 29). This functionality links the sMaf–CncC complex with xenobiotic or oxidative stress, which demands considerable cellular energy and incurs fitness costs, including reduced fecundity (30, 42). Our study, using *A. aegypti* as a model organism, demonstrates that *AaMafB* is actively expressed during vitellogenesis after a blood meal, acting as both a 20E early gene and a 20E sustaining gene. *Aa*MafB promotes the transcription of *AaVg* and Halloween genes, controlling vitellogenesis by heterodimerizing with *Aa*CncC (*SI Appendix*, Fig. S8). The distinct functioning of the sMaf-CncC heterodimer in reproduction may be due to the predominance of JH in governing vitellogenesis in most insects, such as *Tribolium castaneum*. In contrast, vitellogenesis in Dipteran insects is regulated by 20E (6). Despite the conservation of the sMaf-CncC heterodimer in elevating ecdysteroid levels and reducing JH titers (30, 36, 37), this leads to different outcomes in various insects, namely suppressed *Vg* expression in *T. castaneum* but increased *AaVg* expression in mosquitoes. Thus, in addition to its roles in detoxification and antioxidant responses, the involvement of the 20E-early gene *AaMafB* in boosting fecundity provides further insight into the actions of the steroid hormone 20E in insect egg development.

In the female *A. aegypti* mosquito, *AaMafB* was highly expressed at 12 to 24 h PBM, closely correlating with elevated 20E titers and suggesting a response of *AaMafB* to 20E. CHX did not suppress the 20E-mediated induction of *AaMafB*, implying that 20E activates the *AaMafB* transcription directly. The EcR heterodimerizes with USP in the presence of 20E and acts on the EcRE to directly trigger the transcription of 20E target genes, such as *BrC*, *E74*, and *E75* genes (11–14). Our luciferase reporter assays indicated that the 20E–*Aa*EcR–*Aa*USP complex significantly activated the transcription of *AaMafB*. EMSA further confirmed the specific binding of the 20E–*Aa*EcR–*Aa*USP complex to the predicted EcRE in the promoter of the *AaMafB* gene. Several studies have previously identified the EcRE by analyzing the promoters of 20E early or early-late genes, such as *ecdysone-induced protein 28/29*, *HR3*, and *E75*. These identified EcREs have differential sequence requirements (33, 40, 43). By analyzing the binding capacity of various mutated probes toward the 20E–*Aa*EcR–*Aa*USP complex, our results indicate that the motif (GCACGCTGAAC) determines the binding capacity of EcRE in the promoter of *AaMafB*. These findings demonstrate that *AaMafB* serves as a 20E early-response gene.

20E early or early-late genes regulate various insect physiological processes, such as molting, metamorphosis, and reproduction (6, 44). RNAi-mediated depletion of *Aa*MafB hindered the growth of ovarian follicles and dramatically reduced egg deposition, supporting the involvement of *Aa*MafB in mosquito reproduction. Drastically smaller ovarian follicles are linked to compromised Vg synthesis, secretion, or uptake (45, 46). The decreased expression of *AaVg* and *AaVgR* in response to *Aa*MafB depletion may explain the decreased primary follicles and reduced fecundity. Similar phenotypes, such as decreased follicle size and *AaVg* expression, were observed after RNAi of *Aa*CncC, whose isoform X2 was confirmed to be a heterodimeric partner of *Aa*MafB. A slight decrease in *AaCncC* expression was observed at 12 h PBM and was soon recovered at 24 h PBM. Given that *AaCncC* expression did not sharply increase after a blood meal, we concluded that *Aa*MafB is the dominant factor in active vitellogenesis, with *Aa*CncC as a partner.

The sMaf–CncC heterodimer acts on the ARE to activate the transcription of genes associated with detoxification or antioxidant functions (26, 27). Our luciferase reporter assays demonstrated that the *Aa*MafB–*Aa*CncC heterodimer significantly activates *AaVg* transcription. EMSA further confirmed the specific binding of the *Aa*MafB–*Aa*CncC heterodimer to the predicted ARE in the *AaVg* promoter. By analyzing various mutated probes, we identified the motif (CGTGTGACCTAGC) that determines the binding capacity of the ARE in the *AaVg* promoter. The core ARE sequence (TGACNNNGC) has been previously identified (21, 47). Our results revealed that both the core ARE and the four-nucleotide motif (CGTG) preceding it are crucial as either mutation abolished the probes' binding capacity to the *Aa*MafB–*Aa*CncC heterodimer. Interestingly, individual *Aa*MafB or *Aa*CncC proteins bound to the predicted ARE, and this binding was also dependent on the core ARE and the preceding CGTG motif. The sMaf proteins can form homodimers that bind to the Maf recognition element (MARE, TGCTGACTCAGCA). These homodimers compete with the Nrf2–sMaf heterodimer for binding to the ARE, which is embedded in the MARE (48). Since sMaf proteins lack the canonical activation domain, sMaf homodimers act as negative regulators (48, 49). Nevertheless, overexpression of *Aa*MafB or *Aa*CncC alone did not significantly alter luciferase activity, suggesting that *Aa*MafB or *Aa*CncC homodimers are unlikely to be involved in the regulation of *AaVg*. This observation is consistent with findings in *T. castaneum*, where no effect of Maf homodimers on *CYP* gene regulation was noted (28).

Vitellogenesis, a central event in female reproduction, is regulated by 20E (6). Although the 20E-bound EcR–USP heterodimer can directly initiate *Vg* expression (17), several 20E early and early-late genes act synergistically with EcR–USP to amplify *Vg* expression. Binding sites for E74B, BrC-Z2, and βFTZ-F1 have been identified in the *Vg* promoter, and their roles in *Vg* activation have been elucidated (18–20, 50). E74B or BrC-Z2 alone has minimal effects on the *Vg* promoter but enhances *Vg* expression when interacting with the EcR–USP complex (18, 19). βFTZ-F1 facilitates *Vg* expression by forming an interaction with FISC, which aids the assembly of both FISC and EcR–USP on the *Vg* promoter (20). Unlike E74B, BrC-Z2, and βFTZ-F1, the *Aa*MafB–*Aa*CncC heterodimer directly activates the *AaVg* promoter without interacting with *Aa*EcR–*Aa*USP, as demonstrated by luciferase reporter assays using an *AaVg* promoter fragment. However, whether this synergistic action occurs under physiological conditions requires further investigation.

Significantly, *Aa*MafB and *Aa*CncC enhance mosquito reproduction by promoting the transcription of Halloween genes, thereby elevating E and 20E titers. *Aa*MafB and *Aa*CncC target AREs in the *AaShd* promoter, initiating transcription that facilitates E conversion to 20E (38). Ectopic 20E application partially alleviates ovarian deficits due to *Aa*MafB or *Aa*CncC depletion, supporting the heterodimer’s role in maintaining 20E levels during vitellogenesis. Thus, *Aa*MafB influences vitellogenesis through two potential mechanisms: directly regulating *AaVg* expression and indirectly sustaining high 20E levels, which enhances its expression, facilitating vitellogenesis. Consequently, *Aa*MafB modulates the scale and timing of *AaVg* expression after a blood meal. Given the 20E crucial role in post-meal reproduction regulation, *Aa*MafB influence extends further. Reduced *AaVgR* expression in response to *Aa*MafB depletion could result from decreased 20E levels, as E74 and BR-C binding sites are identified in the *VgR* promoter (51). Similarly, *AaMafB* reduction following *AaCncC* depletion may stem from lowered 20E levels. However, the diminished expression of *AaCncC* in response to *AaMafB* depletion may be due to other mechanisms, as *AaCncC* expression does not respond to 20E.

In conclusion, we identified *AaMafB* as a 20E-regulated early gene and mapped 20E-receptor complex’s binding sites within the *AaMafB* promoter. The transcription factor *Aa*MafB, in conjunction with *Aa*CncC, activates the expression of *AaVg* and Halloween genes, thus sustaining high 20E levels and regulating vitellogenesis (*SI Appendix*, Fig. S8). Our study elucidates the binding sites of the *Aa*MafB–*Aa*CncC heterodimer within the *AaVg* and *AaShd* promoters. This research enhances our comprehension of the endocrine regulatory mechanisms underlying insect egg development.

## Materials and Methods

Detailed materials and methods used in this study are described in *SI Appendix*, *SI Materials and Methods*. In vitro FB culture, dsRNA-mediated gene silencing, IB, Co-IP assay, luciferase reporter assay, EMSA, RT–qPCR, and quantification of E and 20E were performed. Primers used in this study are shown in *SI Appendix*, Table S2.

## Supplementary Material

Appendix 01 (PDF)

## Data Availability

All study data are included in the article and/or *SI Appendix*.
