# Supplementary material for: The ecdysone-induced bZIP transcription factor MafB establishes a positive feedback loop to enhance vitellogenesis and reproduction in the Aedes aegypti mosquito
Source: Proc Natl Acad Sci U S A. 2025 Jan 10;122(2):e2411688122. doi: 10.1073/pnas.2411688122 (PMC11745349; doi:10.1073/pnas.2411688122)
Supplement: Supplementary file 1 — Appendix 01 (PDF) [file pnas.2411688122.sapp.pdf]

Supplementary Information for

The ecdysone-induced bZIP transcription factor MafB establishes a positive feedback loop to enhance vitellogenesis and reproduction in the *Aedes aegypti* mosquito

Jia-Lin Wang, Zi-Qian Zhong, Ya-Zhou He, Jun-Hua Tian, Yu-Feng Wang, and Alexander S. Raikhel

Jia-Lin Wang

E-mail: jlwang@ccnu.edu.cn

Yu-Feng Wang

E-mail: yfengw@ccnu.edu.cn

Alexander S. Raikhel

E-mail: alexander.raikhel@ucr.edu

This PDF file includes:

SI Materials and Methods

Figures S1 to S8

Tables S1 and S2

SI References

SI Materials and Methods

Mosquito rearing

As described previously, A. aegypti mosquitoes were maintained at 27 極C and 86% relative humidity (1). Adult mosquitoes continuously receive water and a 10% sucrose solution via wicks. Four-day-old female mosquitoes were blood-fed on white Leghorn chickens, adhering to the guidelines approved by the Institutional Animal Care and Use Committee at the University of California, Riverside.

In vitro FB culture

We prepared the culture medium and performed *in vitro* FB culture using a previously described method (2, 3). To examine the effect of AAs or 20E on *AaMafB* expression, FB samples from female mosquitoes at 96 h PE were incubated in a culture medium containing either ethanol alone (solvent control), AAs, 10^{-6} M 20E (Sigma), or AAs and 10^{-6} M 20E. To further assess the impact of CHX on *AaMafB* or *AaCncC* expression, FB samples at 96 h PE were incubated in a culture medium containing either AAs, AAs+10^{-3} M CHX, AAs+10^{-6} M 20E, or AAs+10^{-6} M 20E+10^{-3} M CHX, using ethanol as solvent. After 6 h of incubation, FB samples were collected for RT-qPCR analyses.

RNAi in mosquitoes and analysis of OV and fecundity

The dsRNAs of *AaEcR*, *AaMafB*, *AaCncC*, *DmEcR*, and *DmUSP* were synthesized using the MEGAscript kit (Ambion), as per the manufacturer's instructions. The dsRNA targeting bacterial *luciferase* gene was synthesized as a control. Primers used for dsRNA synthesis are listed in Table S2. For gene depletion, 0.6 μg (0.3 μl of 2 μg/μl) of the desired dsRNA was microinjected into the thoraxes of cold-anesthetized female mosquitoes within 6 h PE (newly emerged). Mosquitoes were then allowed to recover for 4 days before blood feeding. In the hormonal rescue experiment, 0.3 μl of 20E (10^{-4} M) or ethanol (solvent control) was microinjected into the thoraxes of *Aa*MafB- or *Aa*CncC-depleted mosquitoes at 12 h PBM. FBs were dissected at 72 h PE, 12 h PBM, or 24 h PBM for RT-qPCR analyses or 20E quantification. OVs were dissected at 24 h PBM for RT-qPCR, 20E quantification, or imaging using a stereomicroscope (Leica M165FC, Heerbrugg, Switzerland). The average follicle size for each treatment was measured in 7–14 individuals per replicate, with three replicates in total. For oviposition, 7–14 mated females at 48 h PBM were kept individually in small cages. The

number of eggs laid per female on moist paper and the number of eggs hatched were counted. Each treatment was repeated three times.

Immunoblotting and Co-IP assay

The open reading frames (ORFs) of *AaEcRb* (4) and *AaUSPb* (5) were previously cloned into the pAFW plasmid fused with a Flag tag (6). The ORFs of *AaMafB* and *AaCncCX2* were amplified and cloned into a pIE2 vector (7) fused with Myc and Flag tags, respectively. *Drosophila* S2 cells, growing in a 6-well plate, were co-transfected with pAFW-*AaEcRb* and/or pAFW-*AaUSPb*, or pIE2-*AaMafB* and/or pIE2-*AaCncCX2* plasmids. Cells were harvested and lysed in a lysis buffer (25 mM Tris-HCl, 150 mM NaCl, 1 mM EDTA, 1% Triton X-100, pH 7.5) supplemented with a protease inhibitor (Roche, Mannheim, Germany). The cell lysate was centrifuged at 12,000×*g* for 30 min to remove cell debris, and total protein was quantified using a BCA protein quantification kit (Vazyme, Nanjing, China). The proteins were separated on 7.5% SDS-PAGE gels and transferred to PVDF membranes (Solarbio, Beijing, China). Membranes were incubated with anti-*Aa*EcR antibody (8), anti-Flag antibody (Sigma), or anti-Myc antibody (Proteintech, Chicago, IL, USA). Anti-β-actin antibody (ABclonal, Wuhan, China) was used as a loading control.

For Co-IP assays, 1000 μl of cell lysate was incubated with 10 μg of the antibody (anti-Myc or anti-Flag) at 4°C overnight. Subsequently, 40 μl of Protein A/G Magnetic Beads (Engibody, Dover, DE, USA) were added to the cell lysate and the mixture was gently rotated for 3 h. The beads were then collected using a magnetic separation rack and analyzed by immunoblotting.

RNAi in S2 cells and luciferase reporter assay

The 5'-upstream regulatory regions of *AaMafB* (nt -1217 to -729), *AaVg* (nt -1023 to -26), and *AaShd* (nt -1600 to -336) were synthesized by GenScript Co. Ltd (Nanjing, China) and subcloned into the pGL4.17 reporter plasmid (Promega, Madison, USA). The pGL4.17-*AaVg* derivatives pGL4.17-*AaVg*ΔARE, pGL4.17-*AaVg*ΔEcRE, and pGL4.17-*AaVg*ΔARE+ΔEcRE were also constructed. Each well of *Drosophila* S2 cells was co-transfected with 100 ng of the desired reporter plasmid and 10 ng of the *Renilla* luciferase reporter plasmid pCobia using FuGENE HD transfection reagent (Promega). For the examination of the 20E–*Aa*EcR–*Aa*USP complex on the *AaMafB* promoter, 1.5 μg of *DmEcR* or *DmUSP* dsRNA was also transfected. Additionally, some wells were co-transfected with pAFW-*AaEcRb*, pAFW-*AaUSPb*, or both.

20E was added to the wells with a final concentration of 10^{-6} M at 42 h post-transfection. For the examination of the *Aa*MafB–*Aa*CncCX2 heterodimer on the *AaVg* or *AaShd* promoters, some wells were co-transfected with pIE2-*AaMafB*, pIE2-*AaCncCX2*, or both. For the evaluation of synergistic activation of the *AaVg* promoter by *Aa*MafB–*Aa*CncC and *Aa*EcR–*Aa*USP, some wells were transfected with pAFW-*AaEcRb*/pAFW-*AaUSPb* and/or pIE2-*AaMafB*/pIE2-*AaCncCX2*. S2 cells were treated with or without 20E (1×10^{-6} M) at 42 h post-transfection. Cells were harvested at 48 h post-transfection, and relative luciferase activities were determined using the dual-luciferase reporter assay system (Promega).

EMSA

We performed EMSA according to previously described methods (9, 10). The probes targeting EcRE and ARE were labeled with [γ-^{32}P] ATP (Promega) and 6-FAM (6-carboxyfluorescein, Sangon, Shanghai, China), respectively. Nuclear proteins were extracted from S2 cells overexpressing *Aa*EcRb-Flag and *Aa*USPb-Flag, or *Aa*MafB-Myc and/or *Aa*CncCX2-Flag using a nuclear and cytoplasmic protein extraction kit (Beyotime, Shanghai, China). The nuclear proteins were incubated with [γ-^{32}P] ATP-labeled probes or FAM-labeled probes for 30 min at room temperature. For the competition assays, a 50-fold amount of unlabeled probe was preincubated with the nuclear extracts before the addition of the labeled probe. For supershift assays, anti-*Aa*EcR antibody or anti-Flag antibodies were preincubated with nuclear extracts from S2 cells expressing *Aa*EcRb-Flag and *Aa*USPb-Flag. Anti-Myc or anti-Flag antibodies were preincubated with nuclear extracts from S2 cells expressing *Aa*MafB-Myc and *Aa*CncCX2-Flag. Rabbit IgG (Proteintech) was used as a control. The DNA–protein complexes were resolved, and the probes were visualized using autoradiography or the SapphireNIR-Q imaging system (Azure Biosystems, Dublin, USA).

RT-qPCR

Various tissues at 72 h PE and 24 h PBM, OVs and FBs from different developmental stages and RNAi experiments, and FBs from *in vitro* culture assays and RNAi plus 20E treatments, were collected for total RNA extraction using TRIzol (Invitrogen, Waltham, USA). After treatment with DNase I (Invitrogen), cDNAs were synthesized from 2 μg of total RNA using SuperScript II reverse transcriptase (Invitrogen). RT-qPCR was performed using iQ SYBR Green Supermix (Bio-Rad). Each measurement was conducted in triplicate, and relative

expression was calculated using the $2^{-\Delta\Delta Ct}$ method. Primers used for RT-qPCR are listed in Table S2.

Ecdysone and 20-hydroxyecdysone measurements

The ecdysone titers were measured using an insect Ecdysone ELISA kit (Kmaels Biotechnology, Shanghai, China) following the manufacturer's protocol. For each biological replicate, fifteen mosquitoes were pooled, with three replicates per treatment. Samples were homogenized in 300 μl PBS and then centrifuged at 12,000×*g* for 10 min. Supernatants were loaded onto a 96-well strip plate precoated with E antibodies, followed by incubation with horseradish peroxidase-conjugated detection antibodies for 1 h at 37°C. After development with 3,3′,5,5′-tetramethylbenzidine, absorbance was measured at 450 nm using a Synergy H4 microplate reader (BioTek, Winooski, USA). E titers were determined from a standard curve.

For 20E quantification, FBs and OVs were collected from *iLuc* control (n=30), *iAaMafB* (n=30 FBs, n=110 OVs), and *iAaCncC* (n=30 FBs, n=70 OVs) female mosquitoes. Samples were homogenized in 200 μl PBS and centrifuged at 12,000×*g* for 10 min. Supernatants were processed using an insect 20E ELISA kit (Kmaels Biotechnology) with the same procedure as for E titers. Absorbance was measured at 450 nm, and 20E titers were calculated based on its standard curve.

Statistical analysis

Statistical analysis was performed using GraphPad Prism 8 (GraphPad Inc., La Jolla, CA, USA). Data are presented as the mean ± SE of three biological replicates. Two-tailed Student's *t*-tests were used to evaluate statistical differences between the two groups. One-way ANOVA with Tukey's multiple comparison test was used to determine statistical differences among multiple groups. A *p-value* < 0.05 was considered statistically significant.

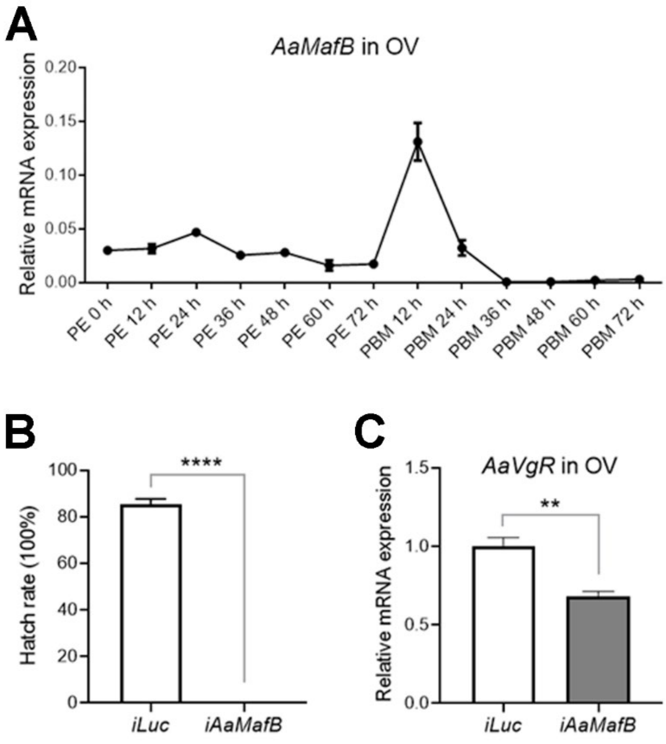

Fig. S1. Depletion of *Aa*MafB decreases egg hatching rate and *AaVgR* expression. (A) Relative mRNA levels of *AaMafB* in the ovary (OV) of *A. aegypti* at various developmental stages. OVs were collected at 0, 12, 24, 36, 48, 60, and 72 h PE, as well as 12, 24, 36, 48, 60, and 72 h PBM. (B) Hatching rate analysis of eggs obtained from *iAaMafB* and *iLuc* mosquitoes. (C) A dramatic decline in *AaVgR* transcripts in *Aa*MafB-depleted OV detected using RT-qPCR. *iLuc*, *luciferase* dsRNA-injected; *iAaMafB*, *AaMafB* dsRNA-injected. Asterisks indicate statistically significant differences determined by a two-tailed Student's *t*-test (**$p < 0.01$ and ****$p < 0.0001$).

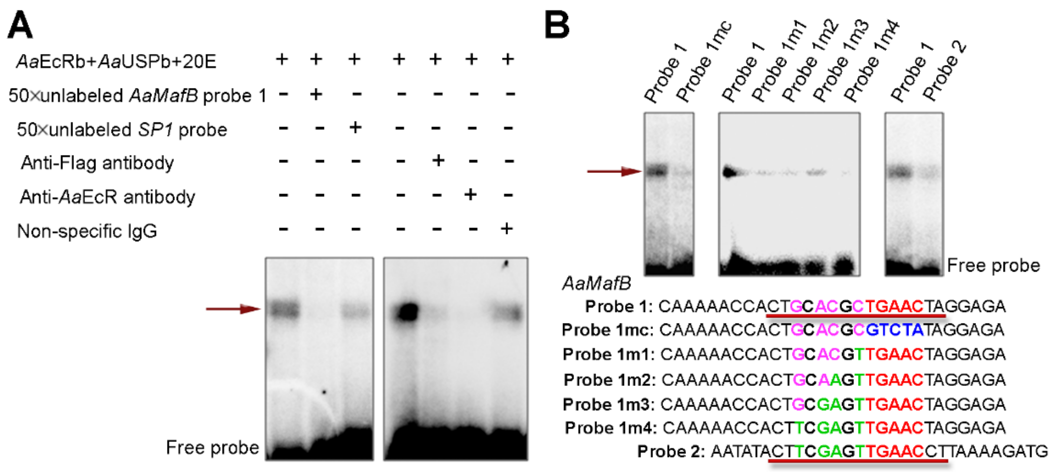

Fig. S2. Characterization of the functional EcRE in the *AaMafB* promoter. (A, B) EMSA confirms the binding of the 20E–*Aa*EcR–*Aa*USP complex to *AaMafB* probe 1 but not probe 2. Probes 1 and 2 were designed to cover EcRE1 (underlined) and EcRE2 (underlined). Mutation of the predicted binding motif of EcRE1 abolished or weakened the binding capacity. Assays were performed using nuclear protein extracts from 20E-treated S2 cells with overexpressed *Aa*EcRb-Flag and *Aa*USPb-Flag fusion proteins. The red arrows represent the specific protein–DNA complex.

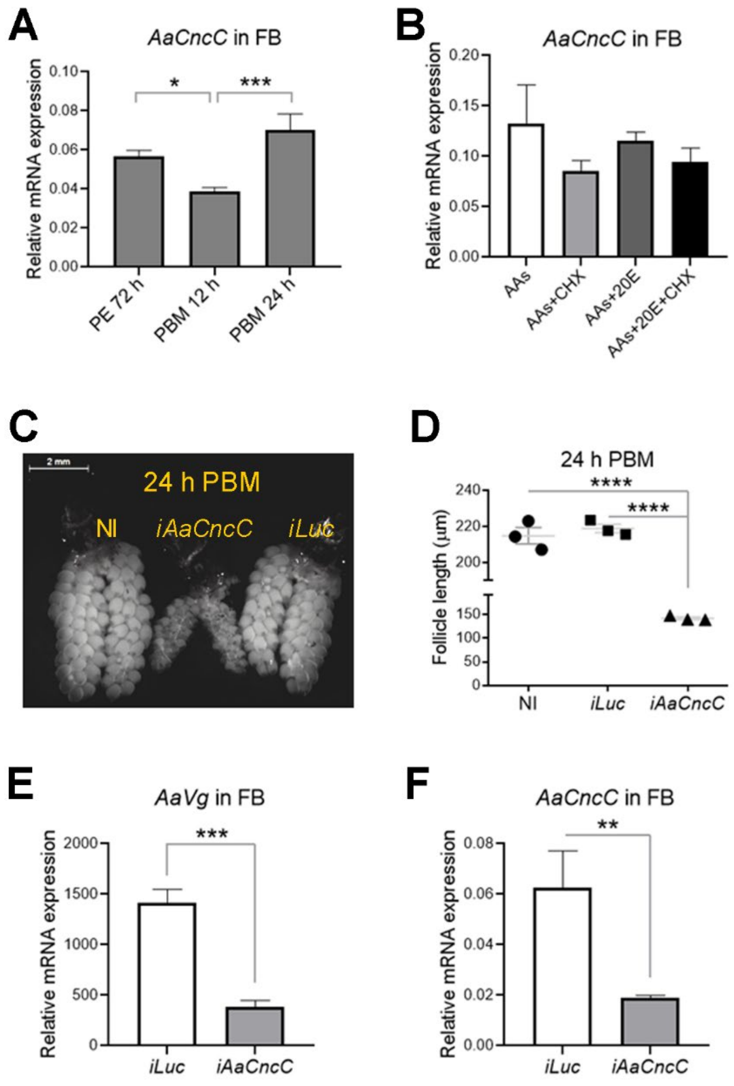

Fig. S3. Depletion of *Aa*CncC decreases follicle size and *AaVg* expression. (A) Relative mRNA levels of *AaCncC* in the FB of female *A. aegypti* at 72 h PE, 12 h PBM, and 24 h PBM. (B) Relative mRNA levels of *AaCncC* in the FB collected from female mosquitoes at 96 h PE and incubated in culture medium containing either AAs, AAs+CHX, AAs+20E, or AAs+20E+CHX, with ethanol as solvent. (C) Representative OVs at 24 h PBM. (D) Average follicle length at 24 h PBM. (E) Dramatically fewer *AaVg* transcripts in *AaCncC*-depleted FB detected by RT-qPCR. (F) RT-qPCR showing the efficiency of *AaCncC* depletion in FB. NI, non-injected; *iLuc*, *luciferase* dsRNA-injected; *iAaCncC*, *AaCncC* dsRNA-injected. Statistical differences were determined using a two-tailed Student's *t*-test or one-way ANOVA test. Asterisks indicate significant differences ($*p < 0.05$, $**p < 0.01$, $***p < 0.001$, and $****p < 0.0001$).

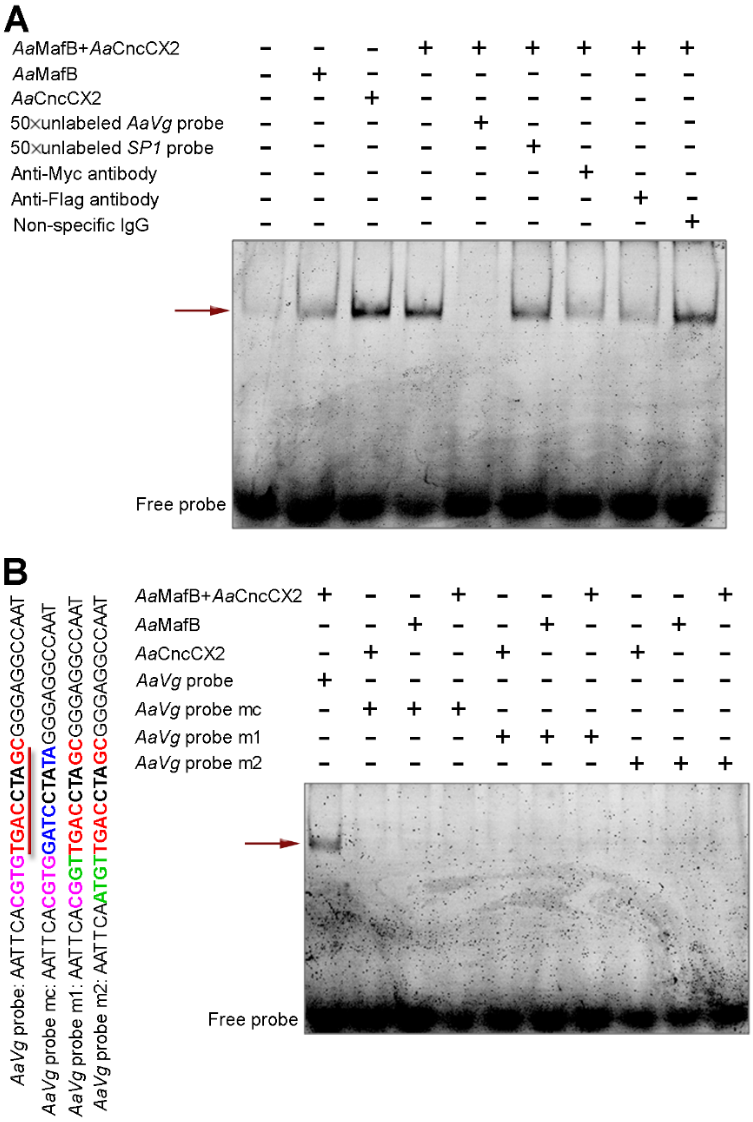

Fig. S4. Characterization of the functional ARE in the *AaVg* promoter. (A, B) EMSA confirming the binding of *Aa*MafB, *Aa*CncCX2, or the *Aa*MafB–*Aa*CncCX2 heterodimer to the *AaVg* probe. The *AaVg* probes containing the core ARE motif (underlined) or the mutated motif are shown. Mutation of the predicted binding motif abolished the binding capacity. Assays were performed using nuclear protein extracts from S2 cells with overexpressed *Aa*MafB-Myc and/or *Aa*CncCX2-Flag fusion proteins. The red arrows indicate the specific protein–DNA complex.

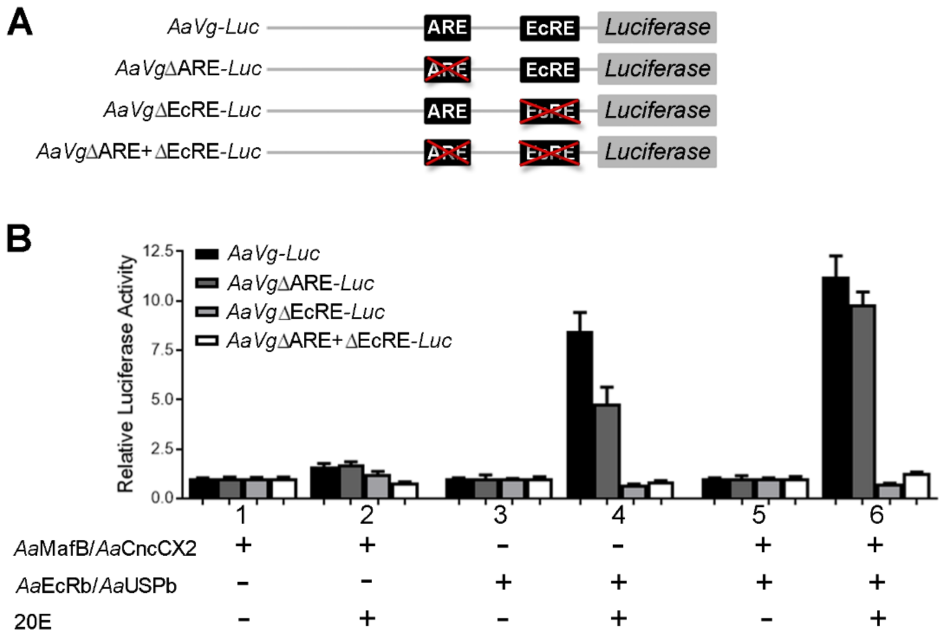

Fig. S5. The lack of synergistic effect of *Aa*MafB–*Aa*CncC with *Aa*EcR–*Aa*USP in activating the *AaVg* promoter. (A) A luciferase reporter construct, pGL4.17-*AaVg*, carrying identified EcRE (11) and ARE sites. pGL4.17-*AaVg*ΔARE, pGL4.17-*AaVg*ΔEcRE, and pGL4.17-*AaVg*ΔARE+ΔEcRE denote pGL4.17-*AaVg* derivatives lacking the functional ARE, EcRE, and both, respectively. (B) Dual-luciferase reporter assay showing no obvious synergistic activation of the *AaVg* promoter by *Aa*MafB–*Aa*CncCX2 and *Aa*EcRb–*Aa*USPb. S2 cells were co-transfected with the reporter vector pGL4.17-*AaVg* (or its derivative) and the overexpression plasmids pIE2/*AaMafB-Myc* and pIE2/*AaCncCX2-Flag*, and/or the plasmids pAFW/*AaEcRb-Flag* and pAFW/*AaUSPb-Flag*. After transfection, S2 cells were cultured with or without 20E (1×10^{-6} M).

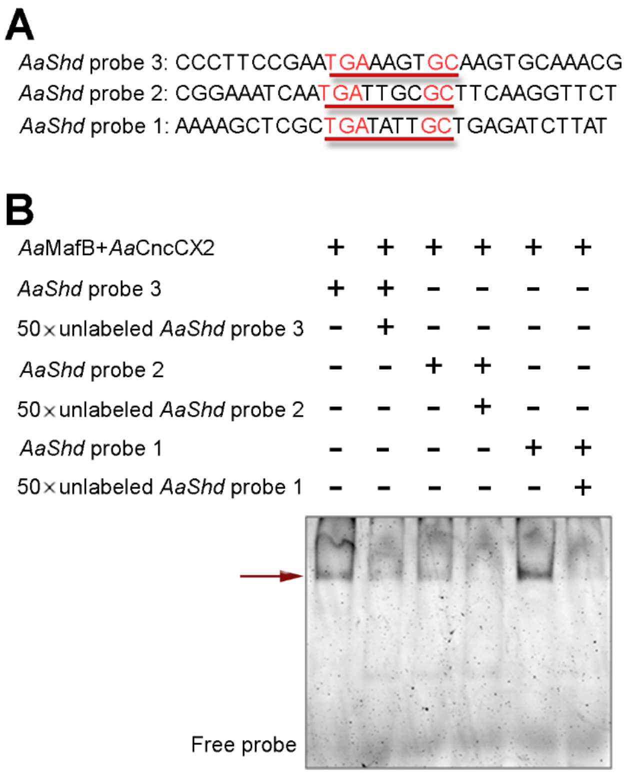

Fig. S6. Characterization of the functional ARE in the *AaShd* promoter. (A) The *AaShd* probes 1, 2, and 3 were designed to cover the ARE1, ARE2, and ARE3 in *AaShd* promoter, respectively. The core ARE motifs are underlined. (B) EMSA confirms the binding of the *Aa*MafB–*Aa*CncCX2 heterodimer to the *AaShd* probes. The binding capacity of *AaShd* probes 1 and 3 was stronger than that of probe 2. Assays were performed using nuclear protein extracts from S2 cells with overexpressed *Aa*MafB-Myc and *Aa*CncCX2-Flag fusion proteins. The red arrow indicates the specific protein–DNA complex.

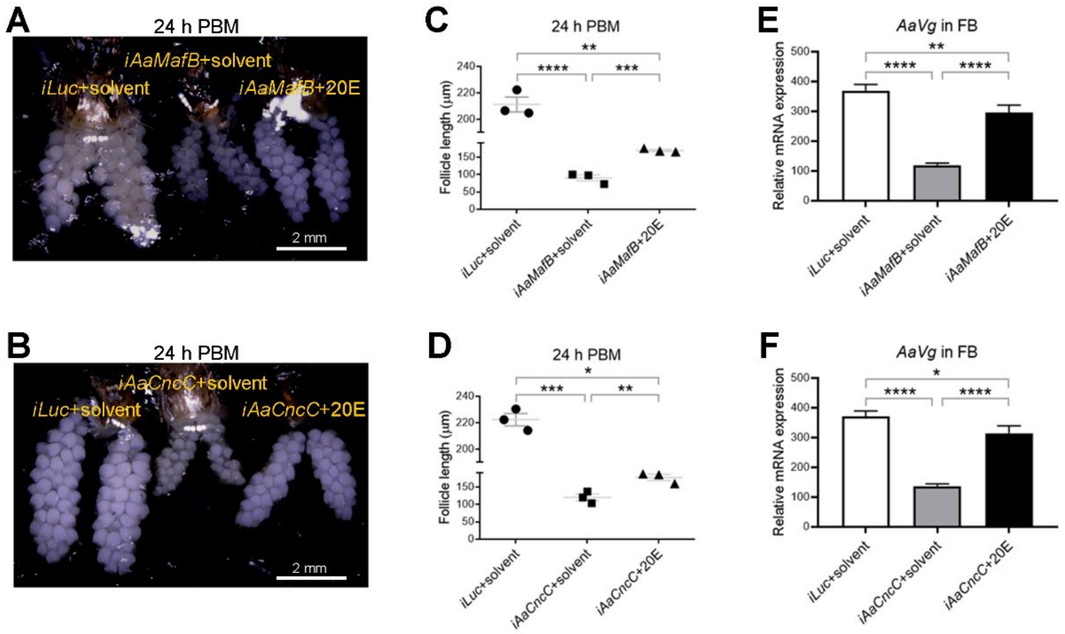

Fig. S7. 20E administration partially alleviates ovarian growth deficits due to *Aa*MafB or *Aa*CncC depletion. (A, B) Representative OVs at 24 h PBM. (C, D) Average follicle length at 24 h PBM. (E, F) 20E treatment upregulated *AaVg* transcript levels in the FB of *Aa*MafB- or *Aa*CncC-depleted mosquitoes at 24 h PBM, as detected by RT-qPCR. *iLuc*+solvent, *luciferase* dsRNA injection followed by ethanol (solvent) injection; *iAaMafB*+solvent/20E, *AaMafB* dsRNA injection followed by ethanol or 20E injection; *iAaCncC*+solvent/20E, *AaCncC* dsRNA injection followed by ethanol or 20E injection. Statistical differences were determined by a one-way ANOVA test. Asterisks indicate significant differences (*$p < 0.05$, **$p < 0.01$, ***$p < 0.001$, and ****$p < 0.0001$).

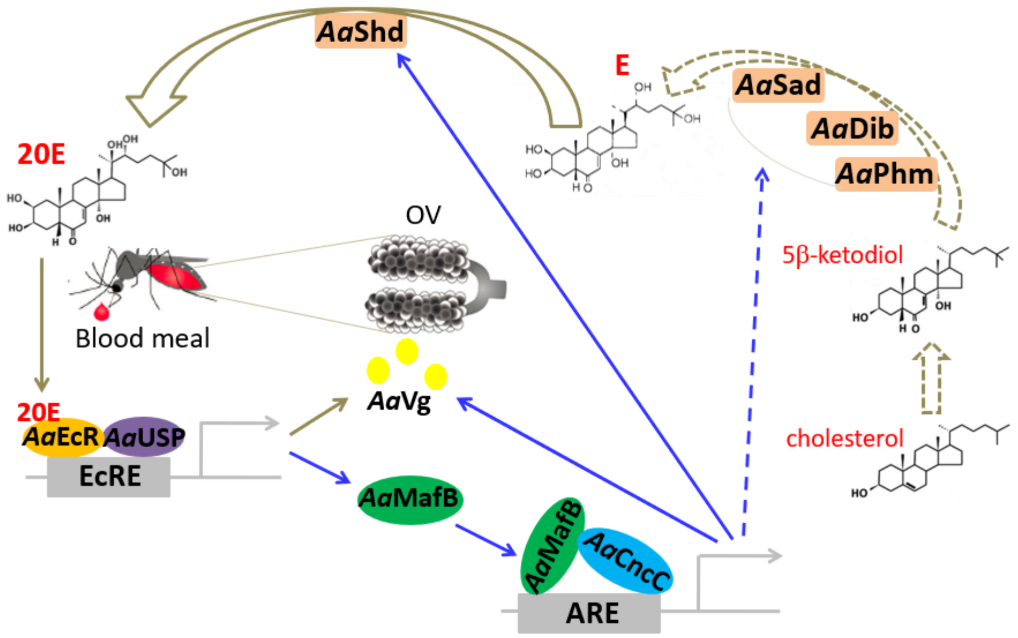

Fig. S8. Schematic diagram of a positive feedback loop mediated by *Aa*MafB, which amplifies 20E signaling and regulates vitellogenesis in the mosquito *A. aegypti*. Following a blood meal, elevated 20E, in conjunction with the *Aa*EcR–*Aa*USP heterodimer, stimulates the expression of *AaVg* and *AaMafB*. *Aa*MafB then heterodimerizes with *Aa*CncC, activating the transcription of *AaVg* and Halloween genes, including *AaPhm*, *AaDib*, *AaSad*, and *AaShd*. Increased levels of *Aa*Phm, *Aa*Dib, and *Aa*Sad facilitate the conversion of 5β-ketodiol to E in the OVs, while elevated *Aa*Shd catalyzes the conversion of E to its active form, 20E, in peripheral tissues. Blue arrows represent the findings from this study, while gray arrows denote results from previous studies (11–13).

Table S1. The predicted Cnc−sMaf binding sites in the promoter of target genes.

Gene	Position		Sequence (5'-3')
	From	To	
AaPhm (*CYP306A1*)	-110	-96	GGTGAGACCGCCAAA
	-332	-318	TTTGAGAGCGCAAAT
	-1056	-1042	GATGAGTCATCATGA
	-1059	-1045	TATGATGAGTCATCA
AaDib (*CYP302A1*)	-942	-928	TATAAGTTAGCATTA
	-1638	-1624	CATGTCGATGCGTAT
AaSad (*CYP315A1*)	-392	-378	AATGAGAAAGCGTTG
	-1272	-1258	GATGATTTAAGAAAA
AaShd (*CYP314A1*)	-395	-381	GCTGATATTGCTGAG
	-879	-865	AATGATTGCGCTTCA
	-1564	-1550	AATGAAAGTGCAAGT

Table S2. List of primers used.

Usage	Target gene	Direction	Primer sequence (5'-3')
RT-qPCR	*AaMafB*	Forward	CCACCTTCATCTCCATGGAC
		Reverse	GCTGCCATGGGCGTAACAAC
	AaCncC	Forward	TTCACCTTGACACCGCTGAC
		Reverse	CCAGCAGTACTTTGAGCTTC
	AaVg	Forward	GCAGGAATGTGTCAAGCGTGAAG
		Reverse	ACGAGGACGAAGAATCGGAAGAG
	AaVgR	Forward	AAGGAGATCACCCGTGCTTG
		Reverse	GTTTCCAGCGTGACCAGGTA
	AaEcR	Forward	CCTCGATGCAGGCCCAGATG
		Reverse	GGCGTTAATTGCTGCCCGTG
	AaUSP	Forward	GCGCGTTCAACCAACAGGTC
		Reverse	ACACAGATGCTTGGACCCGC
	AaShd	Forward	ACCAGAGCGTGCATCAAGG
		Reverse	ACAATACTACCGTCCCAGC
	AaSad	Forward	GAAGGAATCTCTCCGTCTG
		Reverse	TTTACCTCGTCTCTGCCAG
	AaDib	Forward	AATCTCTTCGGCTCAACCC
		Reverse	CTAGATTCTGGGTCACCAC
	AaPhm	Forward	AGAACGGTCGTTCCACTCG
		Reverse	GTGAGGGGTCCATGTGGAC
	AaHR3	Forward	AGGTCGAAGACGAGGTCC
		Reverse	TAACCACCGTGGTGCAGC
	AaE75B	Forward	AAACCGTAATCGCTGCCAG
		Reverse	GTTCTGCGTGCTTTGCTGC
	Aaβ-actin	Forward	TGGACTTGGCTGGTCGCGATC
		Reverse	GCACAGCTTCTCCTTAATGTCAC
	DmEcR	Forward	TAACCATACTCACGGTCCAG
		Reverse	CCATACGCAGCATCATCACC
	DmUSP	Forward	TGGTCCCTATTCCACAGTCC
		Reverse	TCAGCAGAATCACCTGGTCG
	Dmβ-actin	Forward	ACTTCTGCTGGAAGGTGGAC
		Reverse	ATCCGCAAGGATCTGTATGC
dsRNA synthesis	*iAaMafB*	Forward	TAATACGACTCACTATAGGGAGACCACCTTCATCTCCATGGAC
		Reverse	TAATACGACTCACTATAGGGAGAGCGATTGCTGTTGCGAGTGC
	iAaCncC	Forward	TAATACGACTCACTATAGGGAGACACCTTGACACCGCTGAC
		Reverse	TAATACGACTCACTATAGGGAGAGATTCGAGCTCCCTCTCG

	iAaEcR	Forward	TAATACGACTCACTATAGGGACTACAATGCCCTCACCTGC
		Reverse	TAATACGACTCACTATAGGGGGTAAATGCTGGCAGTCCCT
	iDmEcR	Forward	TAATACGACTCACTATAGGGAGAGAACGAGAGCCAAACGGACG
		Reverse	TAATACGACTCACTATAGGGAGATTCTGGTTGCCCAGCGTACG
	iDmUSP	Forward	TAATACGACTCACTATAGGGAGATGGTCCCTATTCCACAGTCC
		Reverse	TAATACGACTCACTATAGGGAGAAGGCAAGCGTACACCTTCTC
	iLuc	Forward	TAATACGACTCACTATAGGGCCTGGATCACTACAAGTACCTCA
		Reverse	TAATACGACTCACTATAGGGCGACAATAGCGTTGGAAAA
Co-IP and Luciferase reporter assay	*AaMafB*-pIE2	Forward	GGGCCGGTACCGCCACCATGGAACTCCCGGCAGAACGC
		Reverse	GATCGCGGCCGCCAGGTCTTCTTCAGAGATCAGTTTCTGTTCTACGTAGTACTCGGGAGA
	AaCncCX2-pIE2	Forward	GGGCCGGTACCGCCACCATGGCGGCATTGGAGTACGAAG
		Reverse	GATCGGGCCCTCACTTATCGTCGTCATCCTTGTAATCCTCTTTCGGTCTATGCTGGC

SI References

1. A. R. Hays, A. S. Raikhel, A novel protein produced by the vitellogenic fat body and accumulated in mosquito oocytes. *Rouxs Arch Dev Biol* **199**, 114-121 (1990).
2. S. G. Roy, I. A. Hansen, A. S. Raikhel, Effect of insulin and 20-hydroxyecdysone in the fat body of the yellow fever mosquito, *Aedes aegypti*. *Insect Biochem Mol Biol* **37**, 1317-1326 (2007).
3. Y. Z. He, E. Aksoy, Y. Ding, A. S. Raikhel, Hormone-dependent activation and repression of microRNAs by the ecdysone receptor in the dengue vector mosquito *Aedes aegypti*. *Proc Natl Acad Sci U S A* **118**, e2102417118 (2021).
4. W. L. Cho, M. Z. Kapitskaya, A. S. Raikhel, Mosquito ecdysteroid receptor: analysis of the cDNA and expression during vitellogenesis. *Insect Biochem Mol Biol* **25**, 19-27 (1995).
5. M. Kapitskaya, S. Wang, D. E. Cress, T. S. Dhadialla, A. S. Raikhel, The mosquito ultraspiracle homologue, a partner of ecdysteroid receptor heterodimer: cloning and characterization of isoforms expressed during vitellogenesis. *Mol Cell Endocrinol* **121**, 119-132 (1996).
6. D. Dong, Y. Zhang, V. Smykal, L. Ling, A. S. Raikhel, HR38, an ortholog of NR4A family nuclear receptors, mediates 20-hydroxyecdysone regulation of carbohydrate metabolism during mosquito reproduction. *Insect Biochem Mol Biol* **96**, 19-26 (2018).
7. P. Xu *et al.*, Expression of recombinant and mosaic Cry1Ac receptors from *Helicoverpa armigera* and their influences on the cytotoxicity of activated Cry1Ac to *Spodoptera litura* Sl-HP cells. *Cytotechnology* **68**, 481-496 (2016).
8. J. Zhu, K. Miura, L. Chen, A. S. Raikhel, AHR38, a homolog of NGFI-B, inhibits formation of the functional ecdysteroid receptor in the mosquito *Aedes aegypti*. *EMBO J* **19**, 253-262 (2000).
9. J. L. Wang, T. T. Saha, Y. Zhang, C. Zhang, A. S. Raikhel, Juvenile hormone and its receptor methoprene-tolerant promote ribosomal biogenesis and vitellogenesis in the *Aedes aegypti* mosquito. *J Biol Chem* **292**, 10306-10315 (2017).
10. A. Yan *et al.*, DAZL regulates proliferation of human primordial germ cells by direct binding to precursor miRNAs and enhances DICER processing activity. *Nucleic Acids Res* **50**, 11255-11272 (2022).
11. D. Martín, S. F. Wang, A. S. Raikhel, The vitellogenin gene of the mosquito *Aedes aegypti* is a direct target of ecdysteroid receptor. *Mol Cell Endocrinol* **173**, 75-86 (2001).
12. T. P. Yao, W. A. Segraves, A. E. Oro, M. McKeown, R. M. Evans, *Drosophila* ultraspiracle modulates ecdysone receptor function via heterodimer formation. *Cell* **71**, 63-72 (1992).
13. R. Lafont, C. Dauphin-Villemant, J. T. Warren, H. Rees, "4 - Ecdysteroid chemistry and biochemistry" in *Insect Endocrinology,* L. I. Gilbert, Ed. (Academic Press, San Diego, 2012), pp. 106-176.
